# Effects of a Capsaicin-Based Phytogenic Solution on Intestinal Permeability, Serum Amino Acid Concentrations, and Digestibility in Heat-Stressed Growing Pigs

**DOI:** 10.3390/ani15121757

**Published:** 2025-06-14

**Authors:** Miguel Cervantes, Panagiotis Sakkas, José A. Valle, Néstor Arce, Ernesto Avelar, Nicolas Quilichini, Adriana Morales

**Affiliations:** 1 Instituto de Ciencias Agrícolas, Universidad Autónoma de Baja California, Mexicali 21100, Mexico; miguel_cervantes@uabc.edu.mx (M.C.);; 2 Laboratory of Nutrition, Faculty of Veterinary Medicine, Aristotle University, 54124 Thessaloniki, Greece; psakk@vet.auth.gr; 3 CCPA Group, Z.A. du Bois de Teillay, 35150 Janzé, France

**Keywords:** heat stress, growing pigs, ileal amino acid digestibility, gut permeability, serum amino acids, *Capsicum* spp., phytogenics

## Abstract

A *Capsicum* spp.-based phytogenic solution (PHY) improved the performance and thermal tolerance of heat-stressed (HS) growing pigs. Two trials were conducted to further evaluate the HS pig response to supplemental PHY. Trial 1: The effects on the serum concentrations of amino acids (AAs) and the gene expression of tight junction proteins in the jejunum and ileum were assessed with 42 pigs (Landrace-Hampshire-Duroc; 27.0 ± 4.5 kg BW). There were three treatments (14 replicates): pigs under thermoneutral (TN) conditions fed control diet (TN-C); and HS pigs fed control diet without (HS-C) or with PHY (HS-PHY). Trial 2: Two-period digestion trial with eight ileal-cannulated pigs to analyze apparent ileal digestibility (AID) of AAs. Period 1: All TN pigs, fed the control (TN-C) or PHY-supplemented (TN-PHY) diet. Period 2: All HS pigs, fed the same diet as in period 1 (HS-C and HS-PHY). The control diet was based on wheat–soybean meal. In the jejunum, HS-C pigs had reduced occludin gene expression (*p* < 0.01) compared to TN-C pigs. HS-PHY pigs increased claudin-2 and tight-junction-protein-1 gene expression compared to HS-C (*p* < 0.05). In the ileum, HS-C and HS-PHY pigs had reduced occludin gene expression (*p* < 0.01). The serum concentrations of AAs decreased in HS-C compared to TN-C pigs (*p* < 0.05); except for arginine and isoleucine, HS-PHY pigs partially recovered serum AA levels. HS tended to reduce (*p* < 0.10; lysine and methionine) and reduced (*p* < 0.05) the AID of essential AAs. PHY did not mitigate the HS-associated reduced AA digestibility. In conclusion, these results support the concept that a phytogenic solution improves intestinal integrity and partially restores the post-absorption metabolism of amino acids, independent of the amino acid digestibility of heat-stressed pigs.

## Simple Summary

Heat stress causes considerable damage to the production parameters of pigs. A previous report showed that a diet supplemented with a phytogenic solution containing *Capsicum* spp. was shown to enhance their thermal tolerance, resulting in increased nutrient consumption and more efficient nutrient utilization. Improved pig performance was associated with lowered body temperatures, enhanced heat-stress defenses, preservation of intestinal integrity, and improved post-absorptive metabolism. This study investigated whether the observed effects are associated with altered amino acid concentrations in blood, the expression of proteins regulating intestinal permeability, and improved amino acid digestibility to further elucidate the mechanism of action. Our findings showed that supplementation improved markers of intestinal permeability and partially restored amino acid concentrations, indicating improved post-absorptive metabolism. However, amino acid digestibility remained unaffected, suggesting that, in conjunction with the results of our previous study, the improved performance is related to enhanced intestinal health, antioxidant status, post-absorptive metabolism, and thermoregulatory responses at a higher level of feed intake. Overall, the tested dietary phytogenic solution may help pigs minimize some of the negative effects of heat stress by operating at multiple levels.

## 1. Introduction

Increased ambient temperatures induce heat stress (HS), which has detrimental effects on pig performance. Pigs exposed to ambient temperature (AT) above thermal neutrality experience increments in their body temperature of up to 2.5 °C [1,2], inducing anorexia aimed at decreasing heat production associated with nutrient metabolism, thus adversely affecting productivity [3]. Concurrently, redirecting blood flow to external organs to enhance heat removal [4] reduces the availability of nutrients and oxygen to the small intestine, causing epithelial damage and shortened intestinal villi [1]. The production of reactive oxygen species (ROS), which damage the epithelia, increases in HS cells. The oxidative stress caused by an elevated concentration of ROS provokes mitochondrial damage, leading to the death of intestinal cells, which, in turn, results in intestinal villus height reduction, compromising the integrity of the small intestine epithelia [1,5,6]. HS-related oxidative stress impairs the small intestine’s digestive–absorptive functions, demonstrated by elevated losses of endogenous amino acids and decreased amino acid digestibility coefficients [7]. This stress response is further associated with the decreased expression of genes encoding amino acid carrier proteins [2] and lower serum concentrations of free amino acids [8]. Additionally, exposure to HS affects the permeability of the intestinal epithelia, as indicated by the reduced expression of tight junction proteins (TJPs) such as claudins, occludins, and ZO-occludin [9], potentially compromising the overall health of HS pigs by increasing endotoxin translocation [10].

Several feeding strategies are proposed to help animals mitigate the effects of HS exposure, including feeding schedule modifications for dairy cattle [11] and changes in diet composition for pigs and poultry, such as reduced fiber and protein content [12,13] and substitution of protein-bound amino acids (AAs) with free AAs [14]. Furthermore, the dietary inclusion of probiotics and phytochemical compounds [15,16], among others, to counteract the negative impact of HS has been studied. Specifically, the effects of HS can be partially mitigated through the use of phytogenics containing plant secondary metabolites [17]. In our prior study [18], dietary supplementation (0.20%) with a *Capsicum* spp.-based phytogenic solution (PHY) was shown to mitigate HS-induced anorexia, enhanced weight gain and feed conversion ratio, reduced body temperature (BT) during peak heat periods, increased serum activities of superoxide dismutase and catalase, and increased intestinal villus height in the jejunum of growing pigs, suggesting protective effects on intestinal integrity and enhanced nutrient absorption with growing pigs.

Besides acting as a thermal and oxidative stress regulator [19], capsaicin, the principal plant secondary metabolite found in peppers, has been found to have marked effects on nutrient digestibility in rats, regardless of the presence of HS [20,21]. These effects have been attributed to enhanced digestive enzyme activities and improved intestinal integrity. Capsaicin, at a dose of 100 µM, has been shown to reduce inflammatory signaling in porcine intestinal epithelial cells, thereby preventing the degradation of TJPs like occludin and claudin, which are critical for maintaining intestinal barrier integrity [22]. Its effects on intestinal permeability, along with increased absorptive surface area in growing pigs [18,19] and increased digestive enzyme activities [23], may result in increased nutrient digestibility when offering capsaicin-based phytogenics. Collectively improved AA digestion absorption and utilization in pigs, which are influenced by both inflammation and HS [8], may restore serum AA concentrations, thereby improving post-absorptive metabolism.

Building on these findings, the current study aims to further explore the impacts of a *Capsicum* spp.-based phytogenic solution on intestinal function by measuring the expression of genes coding for tight junction proteins in the jejunum and ileum, such as occludin, claudin-2, and tight junction protein-1, which serve as markers of intestinal permeability. We further assessed the concentrations of amino acids to reveal whether the supplementation of the capsaicin-based phytochemical is capable of maintaining their concentration in the face of heat stress. Finally, we carried out a digestibility trial to determine whether the observed effects of this phytochemical supplementation were associated with an improvement in amino acid digestibility *per se*.

## 2. Materials and Methods

### 2.1. General

The methodology and materials of the two experiments are presented sequentially. In both experiments, the Official Mexican Regulations on Animal Care [24] guidelines were followed. The Ethics Committee of our institution approved the use of the animals in both experiments (#Dirección/ICA/268/2024-1). Experiments were conducted at the Metabolic-Physiology Unit in northwest Mexico during July and August, when the highest ATs are recorded every year. In both trials, pigs were accommodated either in thermoneutral (TN) conditions inside an air-conditioned room (22 ± 2 °C) or inside a room without air conditioning. Hygrothermographs (Thermotracker Inc., Whitewater, WI, USA) located inside the rooms measured and recorded the AT and relative humidity every 60 min. With these parameters, the temperature–humidity index was calculated following the mathematical model proposed by Rothfusz [25].

### 2.2. Experiment 1

#### 2.2.1. Animals, Diet, and Experimental Procedure

Readers are referred to our previous publication for further details regarding animal housing, diets, and experimental procedures of Experiment 1 [18]. Briefly, forty-two crossbred (Landrace × Hampshire × Duroc) pigs (27.0 kg average initial BW) were randomly allotted to three treatment groups based on a complete block experimental design, considering sex, initial BW, and litter as the main criteria. There were 14 replicates per treatment (7 males and 7 females). Treatment 1 was a group of pigs exposed to TN conditions that were offered a control diet (TN-C). Treatments 2 and 3 were two groups of pigs housed inside the room with no air conditioning (HS room) and were offered the same control diet, either lacking (HS-C) or including the additive (HS-PHY). The control diet (Table 1) consisted of wheat and soybean meal as the major feedstuffs, added with sufficient crystalline Lys, Met, and Thr that met or exceeded the SID AA recommendations for early growing pigs (25–50 kg BW) by NRC [26]. The HS-PHY diet resulted from supplementing 2 g/kg of PHY to the control diet. Capsaicin was the principal active PSM in the PHY containing *Capsicum* spp. oleoresin (trade name ThermoControl^®^, CCPA Group, Janzé, France), but the exact content is not disclosed by the manufacturer. Zhao et al. [22] reported the best response of porcine intestinal cells to 100 µM of capsaicin, which is estimated to be close to the dose employed in this study, assuming at least half of PHY is capsaicin. Pigs were allowed ad libitum access to feed and water all the time. Simultaneously, 12 male pigs with cannulae in the terminal ileum (4 pigs/treatment) were implanted with thermographs (Thermotracker Inc.) to record BT inside the small intestine milieu at 15 min intervals throughout both experiments. Pigs had 10 days of adaptation to the experimental pens under TN environment; following, pigs were randomly allotted to their experimental diet and AT conditions during 8 days; the first 8 days of exposure to HS are the most critical for growing pigs [1]. Six pigs per treatment, closest to the average body weight, were fasted for 10 h starting at 2100 on day 8. On day 9 at 0700, each pig received 500 g of their corresponding feed. At two hours post-feeding, they were sacrificed by exsanguination following electrical stunning for sample collection. The collection of blood samples, which were used for analyzing the concentrations of free essential amino acids (EAAs) and non-essential amino acids (NEAAs) in serum (SC), was accomplished when the animals were being exsanguinated. Serum was separated from blood cells after centrifugation of blood samples at 1000× *g* and 4 °C for 1 min. Jejunum and ileum mucosa were scratched using a glass slide, collected into 2 mL containers, and immediately stored in liquid nitrogen. The total collection process was completed within 10 min, ensuring high-quality materials for the extraction of RNA. Samples were stored at −82 °C pending analysis.

#### 2.2.2. Serum Concentration (SC) of Amino Acids

The SC of free AAs was analyzed according to Sunde et al. [27]. Serum was deproteinized with a Millipore Ultrafree-MC 10,000 NMWL Filter Unit (Millipore, Bedford, MA, USA) and centrifuged at 5000× *g* and 4 °C for 30 min. The amino acid content in feed ingredients, diets, and serum was determined at the University of Missouri Chemical Labs.

#### 2.2.3. Gene Expression

##### Extraction and Purification of Total RNA

Mucosal samples from the jejunum and ileum were pulverized into liquid nitrogen, and total RNA was extracted using the Trizol reagent (Invitrogen, Corp., Waltham, MA, USA) and an RNA purification kit (Direct-zol RNA Microprep, Zymo Research, Irvine, CA, USA), following the instructions from the manufacturer. The concentration of RNA was determined spectrophotometrically (Genezys 50, Thermo Scientific Co., Rochester, NY, USA), and purity was assessed by using the A260/A280 ratio, which fluctuated from 1.8 to 2.0. The integrity of RNA was observed by 1% agarose gel electrophoresis; for each RNA, the 28S:18S rRNA ratio was nearly 2.0:1 [28]. Purified RNA was stored at −82 °C.

##### Reverse Transcription

Before reverse transcription, 2 μg of RNA was treated with DNase I (1 U/µL; Invitrogen) in a 30 μL reaction. The reverse transcription reactions contained 2 µL of DNase-treated RNA, 1 µL of dNTPs solution (10 µM each), 1 µL of random primers (150 ng/µL, Invitrogen), 1 µL of ribonuclease inhibitor (40 U/µL; RiboLock, Thermo Scientific), 2 µL of 5× reverse transcription buffer, 1 µL of reverse transcriptase enzyme (200 U/µL; RevertAid, Thermo Scientific), and 2 µL of nuclease-free water. The reaction was carried out by incubating at 42 °C for 50 min. The reaction was stopped by incubating for 15 min at 70 °C, and then immediately cooling on ice. The cDNA was quantified by spectrophotometry and diluted to a concentration of 50 ng/µL.

##### Quantitative PCR

Primers for three TJPs (claudin-2, occludin, and TJP1) and actin were designed according to their mRNA sequences published in GenBank (Table 2). Actin was the endogenous control used to normalize deviations in mRNA. Quantitative PCR (qPCR) assays were performed to estimate the gene expression of TJP using the specific primers and the Maxima SYBR Green/ROX qPCR Master Mix (Thermo Scientific) in a CFX96 Real Time System thermal cycler (Bio-Rad, Herefordshire, UK) and using the software CFX Manager 3.0 (BioRad).

### 2.3. Experiment 2

#### 2.3.1. Animals, Diet, and Experimental Procedure

Eight male pigs (Landrace × Hampshire × Duroc) adapted with cannulae implanted at the end of the small intestine [29] were used in a digestion trial. The pigs were housed in individual cages. After surgery recovery, two groups of 4 pigs each were created. The trial was conducted in two periods of 8 days each, and, in each period, the two groups of pigs were assigned at random to two diets: a control diet without or with the additive PHY. In period 1, all pigs were accommodated under the TN environment, whereas in period 2, the accommodation of all pigs was in HS conditions; period 2 followed immediately upon the termination of period 1. There were four treatments: TN pigs offered the control diet (TN-C) or the control diet supplemented with the additive (TN-PHY), and HS pigs offered the diet without (HS-C) or with the additive (HS-PHY). The trial was conducted as a completely randomized experimental design with a 2 × 2 factorial (AT and supplemental PHY) arrangement. The ingredients and calculated chemical composition of the experimental diets were the same as those used in Experiment 1 (Table 1). Each 8 d period included the following: 6 days of adaptation to the diet, followed by a 2-day phase for the collection of digesta. The adaptation phase of period 1 (TN conditions) was aimed at training all pigs to ingest the same amount of feed in 30 min or less, equivalent to that of the pig with the smallest ingestion. Pigs exposed to HS, from our previous observations, consume approx. 30% less feed than TN ones. Thus, all pigs in period 1 received their daily meals, whose size was equivalent to that of the pig with the smallest amount of feed ingested during the training, and we deducted the 30% observed in warm environments. In period 2, there was a 10% increment in the size of the meals offered to all pigs. This strategy was designed to ensure that both TN and HS pigs had the same consumption. All pigs were fed two times every day, at 0700 and 1900 h, for the complete experiment. Feed was mixed with water at a 1:1 ratio. Water was provided to all pigs between meals. In each period, ileal digesta was collected in plastic bags attached to the cannula barrel for 12 consecutive hours, beginning at 0700 h on the 7th and 8th days. The bags stayed attached for up to 15 min or less. For each pig and within a specific day of collection, the ileal content was pooled and immediately stored at −20 °C. Samples of digesta were thawed, homogenized, subsampled, and freeze-dried prior to their lab analyses. An external indigestible marker (chromic oxide at 0.2%) was added to the diet to calculate the AA digestibility coefficients.

#### 2.3.2. Chemical Analyses and Calculations

Dry matter, crude protein (CP; method 984.13 A–D), and AA (method 982.30 E) analyses were performed in feed and dried digesta samples milled through a 1 mm screen in a Wiley mill (Thomas Scientific, Swedesboro, NJ, USA). The AA analysis was performed by HPLC (method 982.30 E), with derivatization performed post-column with ninhydrin, and a fluorescence detector was used. Trp was not determined. The analysis of chromic oxide was also performed according to Fenton and Fenton [30], using spectrophotometry. The calculated values for the AID of each AA (%) were achieved using the chromic oxide coefficients.

### 2.4. Statistical Analysis

Analyses of variance of the data were performed based on the experimental design using Statistix 10 software. For the 1st experiment, the effects of AT and PHY on HS pigs were tested with the following contrasts: 1, TN-C vs. HS-C; and 2, HS-C vs. HS-PHY, respectively, based on a complete block experimental design. The 2nd experiment was conducted as a completely randomized design with a 2 × 2 factorial arrangement, with AT and PHY supplementation as the main factors. The effects of AT, PHY, and their interaction were tested as follows: AT (TN-C and TN-PHY vs. HS-C and HS-PHY); supplemental PHY (TN-C and HS-C vs. TN-PHY and HS-PHY); and AT x PHY interaction. Differences, using Student’s t-tests, were considered as significant or as tendency when *p* ≤ 0.05 or when *p* > 0.05 but ≤0.10, respectively.

## 3. Results

### 3.1. Serum Concentration of Free Essential Amino Acids

The ambient temperature significantly affected the SC of EAAs (*p* < 0.05), except for isoleucine, with HS-C pigs exhibiting lower values compared to TN-C pigs (Table 3). Apart from arginine and isoleucine, PHY supplementation significantly increased (*p* < 0.05) the SC of EAAs in HS pigs. For NEAAs, HS-C pigs had a significantly lower SC of asparagine, glutamine, proline, serine, and tyrosine (*p* < 0.05), while alanine tended to reduce (*p* < 0.10), when compared to TN-C pigs. However, HS-PHY pigs had a significantly greater SC of alanine, asparagine, glutamine, proline, serine, and tyrosine compared to HS-C pigs (*p* < 0.05).

### 3.2. Tight Junction Proteins in Jejunum and Ileum

**Figure 1:**
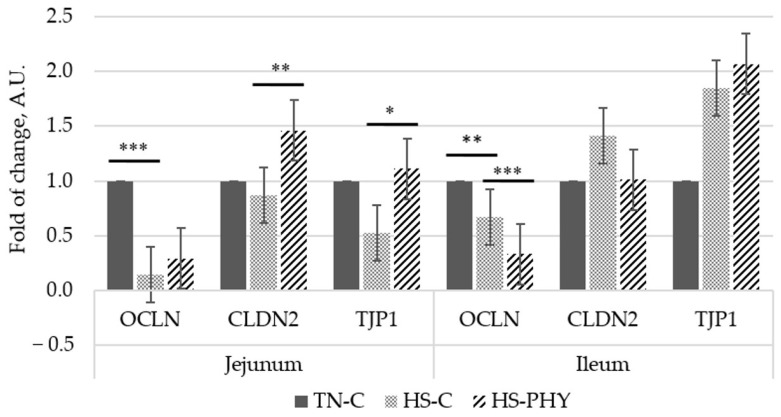
Relative expression of occludin (OCLN), claudin-2 (CLDN2), and tight junction protein-1 (TJP1) in jejunum and ileum. Thermal neutral pigs offered the control diet (TN-C), or heat-stressed pigs offered the control without (HS-C) or with the phytogenic solution (HS-PHY). Contrasts: AT, TN-C vs. HS-C; PHY, HS-C vs. HS-PHY; AT-PHY, TN-C vs. HS-PHY. *** *p* < 0.001; ** *p <* 0.05; * *p <* 0.10.

In the jejunum, HS-C pigs exhibited a significant decrement in occludin gene expression compared to TN-C (*p* < 0.01), but no difference was observed when compared to HS-PHY (Figure 1). Claudin-2 gene expression increased (*p* < 0.05) and that of TJP1 tended to increase (*p* < 0.10) in HS-PHY pigs as compared to HS-C. In the ileum, both groups of HS pigs (HS-C and HS-PHY) had reduced gene expression of occludin in comparison with the thermal neutral pigs (*p* < 0.05); HS-PHY pigs had lower occludin expression than HS-C ones (*p* < 0.01). Neither AT nor PHY had a significant effect on the expression of claudin-2 and TJP1 in the ileum (*p* > 0.10).

### 3.3. Apparent Ileal Digestibility (AID) of Essential and Non-Essential Amino Acids

Heat-stress conditions significantly reduced the AID of all EAAs and crude protein (*p* < 0.05) except for methionine and lysine, which showed a tendency to decrease (*p* < 0.10) in comparison with pigs exposed to the TN environment (Table 4). Phytogenic supplementation did not improve the AID of crude protein or EAAs. For NEAAs, heat stress decreased the AID of glutamate, proline, and serine (*p* < 0.05). In contrast, the AID of alanine, aspartate, cysteine, glycine, and tyrosine was not affected by environmental conditions (*p* > 0.05). Herbal extract supplementation had no significant effect on the AID of any NEAA; however, under TN environments, the AID of cysteine and glycine in TN-PHY pigs tended to be higher than in TN-C pigs (*p* < 0.10).

## 4. Discussion

The AT range inside the HS housing during the experimental periods (29.8 to 35.1 °C) in combination with the relative humidity recordings resulted in a temperature and humidity index equivalent to up to 111. This index was well above that of the comfort zone upper limit (around 80) for pigs. In contrast, the AT recorded inside the TN housing (22.6 to 25.2 °C) combined with the relative humidity produced an index lower than 78. In agreement with a previous report [18], the BT fluctuations of pigs in the HS housing (39.6 to 41.2 °C) seemed to mimic the AT changes, in contrast with TN pigs, whose BT stayed comparatively constant at around 39.5 °C. Likewise, feed intake and the voluntary physical activity of HS pigs showed a dramatic decrement compared to TN pigs. The combined responses of pigs inside the HS housing clearly indicate that these pigs experienced HS conditions during the trial.

Exposing pigs to HS provokes multiple physiological, metabolic, and behavioral adjustments, which negatively impact their productive parameters. The initial 7 days of exposure to warm environments or acute HS [1,31] are highly critical and have the greatest impact. Reduced voluntary feed intake is associated with a lower blood flow to internal organs, as reported previously [32], and inevitably lowers the delivery of nutrients to the intestinal tissue. As a result, injury to the small intestine epithelium, evidenced by the shortened villus length, is frequently noticed in HS pigs [1,33]. The intestinal villi of the HS pigs used in the current study (previously reported in [18]) were substantially shorter than in the TN pigs, which translates into a lower number of enterocytes per intestinal villus. Enterocytes, the most abundant cells of the intestinal villi, perform the absorption of amino acids [34] through the activity of specific proteins that selectively transport amino acids from the intestinal milieu to the blood, across the apical and basolateral membranes [35]. Accordingly, some amino acid transporters are less abundant in pigs exposed to high AT [2], which is expected to negatively affect their intestinal capacity to absorb amino acids.

The SC of amino acids reflects their absorption, especially when blood samples are collected at 1.5 to 2.5 h after feeding [36]. In the current study, the concentration of nine essential amino acids in serum from blood collected 2.0 h postprandially decreased from around 20 to 60% in HS pigs compared to TN pigs, even though all pigs consumed a similar amount of amino acids. This response is in line with the assumed reduction in the number of enterocytes per intestinal villus in HS pigs, which is associated with the reported decreased richness of selected amino acid carriers. Thus, the reduced SC of lysine, histidine, and arginine (27, 36, and 25%, respectively) in HS-C pigs, compared to the TN-C, is attributed to the reported lower richness of the amino acid carrier b^0,+^ that is specific for these three amino acids [35]. Likewise, the reduced SC of methionine, phenylalanine, tryptophan, leucine, and valine (53, 41, 25, 19, and 27%, respectively) in the HS-C pigs is attributed to a lower abundance of the transporter B^0^, which is specific for neutral amino acids [35]. Threonine was the amino acid with the largest SC reduction, around 60%. Mucin, the main component of the intestinal mucosal barrier, contains around 34% threonine [37], which explains the considerably low SC of threonine. Likewise, the SC of proline and serine, two non-essential amino acids also abundant in mucin (15 and 12%, respectively), was reduced to around 35% in HS pigs. Mucin synthesis increases in pigs exposed to HS [1], and this increment has been linked to the elevated production of ROS in HS pigs. Thus, the reduced SC of those amino acids suggests that higher amounts of threonine, proline, and serine are used in the intestinal cells of HS pigs to synthesize mucin, such that lower amounts of these amino acids are transferred to systemic blood.

In our previously published study [18], a phytogenic solution (PHY) containing *Capsicum* spp. mitigated the negative impacts of HS on pigs by (i) lowering the body temperature, (ii) improving the antioxidant status resulting from the increased activity of two powerful antioxidant enzymes (superoxide dismutase and catalase), and (iii) partially restoring the height of jejunum villi and the villi height:crypt depth ratio. Similarly, other reports [22] indicate that capsaicin improved intestinal barrier integrity and glucose absorption by increasing the expression of genes coding for TJP and the glucose transporter SGLT1. We further assessed the effects of the capsaicin-based feed additive on the SC of amino acids, as well as the expression of tight junction proteins in intestinal mucosal samples obtained from our first experiment, to elucidate whether the supplementation of this feed additive affected the absorption of AAs and the richness of tight junction proteins. In support of those findings, HS-PHY pigs increased the SC of eight essential amino acids—arginine and isoleucine were the exceptions—compared to HS-C pigs. Actually, except for arginine and phenylalanine, the SC of essential AAs in the HS-PHY pigs was similar to the SC in the TN-C pigs. Interestingly, the SC of threonine was around 2.3-fold higher in HS-PHY pigs than that of HS-C; proline and serine also increased by about 60% in HS-PHY pigs. In line with the restored jejunal villus height, these increments may suggest an improvement in the stability of the intestinal mucosa, likely attributed to the improved antioxidant environment provoked by the capsaicin-based additive present in PHY. As reported in our previous study, the analyzed antioxidant activity in the PHY-supplemented diet was 2-fold higher than that in the control diet without PHY, which resulted in a 37% increment in the antioxidant activity of digesta collected from HS-PHY pigs. Accordingly, the increased SC of lysine, histidine, methionine, phenylalanine, tryptophan, and valine in HS-PHY pigs, compared to HS-C pigs, indicates an improved absorption capacity of the former pigs. It is worth mentioning the well-recognized function of methionine as an antioxidant [38,39]; thus, the 2.1× increment in serum of the HS-PHY pigs may also suggest a methionine-sparing effect of the capsaicin-based additive. The increments in the SC of amino acids, combined with the higher abundance of the mRNA coding for the claudin-2 synthesis, which is an important component of the small intestine mucosal barrier, additionally support the hypothesis that the capsaicin-based additive may help to improve the stability of the intestinal epithelia.

Intestinal peptidases that are located in the apical membrane of the enterocytes [40] have an active role in complementing the digestion of proteins initiated by pancreatic proteases. Thus, the decreased intestinal villus height provoked by exposing pigs to HS conditions supposedly decreases the abundance of those enzymes, which, in turn, may negatively impact the digestion and absorption of amino acids. Based on that assumption, we also wanted to further assess the effect of adding the capsaicin-based additive to the control diet on the apparent ileal digestibility of pigs exposed to HS. The reduced digestibility of all essential amino acids in HS-C pigs coincides with previous reports [7], which is associated with the reduced intestinal villus height and the assumed reduction in both the number of enterocytes, which, in turn, is expected to reduce the abundance of intestinal peptidases and amino acid transporters. However, adding the capsaicin-based additive to the diet did not improve the digestibility of any amino acid as would be expected because of the improved height in the jejunal villi and the elevation in the SC of amino acids observed in the HS-PHY pigs. Although no clear explanation for this apparent discrepancy can be provided, the ratio between the death of intestinal villus cells and their renewal may help with understanding it. The life span of cells in the intestinal villus ranges from 3 to 5 days [41], but it decreases substantially when pigs are exposed to several stressors, such as high AT (HS), as evidenced by their decreased villus height. Thus, HS pigs appear to increase the proliferation of intestinal cells, which means that a higher amount of amino acids ingested with the diet are utilized for the synthesis of proteins taking place in the newborn cells, resulting in a lower amount of amino acids being transported to the blood. In contrast, the partially restored villus height in the HS-PHY pigs, apparently associated with the improved antioxidant environment in their intestinal digesta, suggests a decreased death rate of intestinal cells. Hence, compared to HS-C pigs, lower intestinal cell proliferation may have occurred in the HS-PHY pigs, suggesting a decreased utilization of amino acids for protein synthesis in the intestinal cells. Nevertheless, supplementary studies are needed to test this hypothesis.

## 5. Conclusions

The results of the present experiment corroborate that pigs exposed to heat stress have lower digestibility and serum concentrations of amino acids, as well as lower expression of tight junction proteins in the jejunum. The supplementation of a phytogenic additive based on *Capsicum* spp. to the diet appears to help pigs, partially counteracting the undesirable effects of acute heat stress on tight junction expression in the jejunum and on serum amino acid concentrations, but not on amino acid digestibility, suggesting that effects on performance are linked with improvements in gastrointestinal integrity, post-absorptive metabolism, and increased feed intake.

## Figures and Tables

**Table 1:** Ingredients and calculated energy and amino acid composition of the experimental diets.

Ingredient	Basal	PHY-Supplemented
Wheat	84.46	84.26
Soybean meal	12	12
L-Lysine.HCl	0.54	0.54
L-Threonine	0.14	0.14
DL-Methionine	0.06	0.06
Phytogenic solution ^1^		0.20
Ca carbonate	1.40	1.40
Di-Ca phosphate	0.65	0.65
Iodized salt	0.35	0.35
Vitamin and mineral premix ^2^	0.40	0.40
Calculated composition, %		
Net energy, MJ/kg	10.10	10.10
Standardized ileal digestible amino acid (SID), %		
SID Arginine	0.85	0.85
SID Histidine	0.38	0.38
SID Isoleucine	0.57	0.57
SID Leucine	1.06	1.06
SID Lysine	0.98	0.98
SID Methionine	0.28	0.28
SID Methionine + Cysteine	0.57	0.57
SID Phenylalanine	0.71	0.71
SID Threonine	0.62	0.62
SID Tryptophan	0.19	0.19
SID Valine	0.66	0.66

^1^ Containing *Capsicum* spp. oleoresin with capsaicin as the principal active (ThermoControl^®^). ^2^ Provided per kg of diet: vitamin A, 4800 IU; vitamin D3, 800 IU; vitamin E, 4.8 IU; vitamin K3, 1.6 mg; riboflavin, 4 mg; D-pantothenic acid, 7.2 mg; niacin, 16 mg; vitamin B12, 12.8 mg; Zn, 64 mg; Fe, 64 mg; Cu, 4 mg; Mn, 4 mg; I, 0.36 mg; Se, 0.13 mg.

**Table 2:** Primers used for the quantitative PCR analyses of mRNA derived from claudin-2, occludin, tight junction protein-1, and actin from pigs.

mRNA	Primer Sequence	Amplicon (bp)
Sus scrofa claudin 2, mRNA (CLDN2, GenBank: NM_001161638.1)		
	Fw 5′AGCTGGCGAACGAGTTCTTA3′	343
	Rv 5′TCCCATGAAGATTCCACGCA3′	
Sus scrofa occludin, mRNA (OCLN. GenBank: NM_001163647.2)		
	Fw 5′AGGCGTCAGGGTCTCTCTAC3′	347
	Rv 5′CTCCGCATAGTCCGAAAGGG3′	
PREDICTED: Sus scrofa tight junction protein 1, transcript variant X1, mRNA (TJP1; GenBank: XM_021098827.1)		
	Fw 5′TGGTATGGGTTTCTGAGGGGA3′	251
	Rv 5′AGGCTCAGAGGACCGTGTAA3′	
Sus scrofa cytoskeletal beta actin mRNA, partial cds (GenBank: AY550069.1)		
	Fw 5′ACAGCAGTCGGTTGGATGG3′	311
	Rv 5′TGCCCACTCAAAATAAACCAAC3′	

**Table 3:** Concentration of free amino acids (µg/mL) in serum of thermal neutral pigs offered the control diet (TN-C) or heat-stressed pigs offered the control without (HS-C) or with the phytogenic solution (HS-PHY).

	Treatment				Contrast *p*-Value ^1^	
Item	TN-C	HS-C	HS-PHY	SEM	AT	PHY
Essential AA						
Arginine	51.4	38.5	42.9	2.6	0.005	0.256
Histidine	11.0	7.0	11.9	0.4	0.001	0.001
Isoleucine	20.0	18.2	19.7	0.9	0.200	0.279
Leucine	28.8	23.4	30.6	1.6	0.039	0.010
Lysine	49.3	36.0	46.6	2.5	0.003	0.012
Methionine	8.5	4.0	8.5	0.6	0.001	0.001
Phenylalanine	19.9	11.7	22.4	0.7	0.001	0.001
Threonine	37.9	15.6	35.5	1.9	0.001	0.001
Tryptophan	12.0	9.0	12.6	0.6	0.004	0.001
Valine	39.9	29.1	42.2	1.6	0.001	0.001
Non-essential AA						
Alanine	104.7	91.1	113.1	5.1	0.083	0.010
Aspartate	5.5	4.6	6.0	0.6	0.347	0.154
Asparagine	15.7	9.2	16.3	1.2	0.002	0.001
Glutamate	36.5	41.3	35.3	4.8	0.502	0.400
Glutamine	101.8	79.8	103.8	6.1	0.026	0.017
Glycine	53.2	61.3	61.3	3.5	0.124	0.994
Proline	84.7	53.8	83.1	5.4	0.001	0.002
Serine	23.4	15.8	25.5	1.8	0.010	0.002
Tyrosine	22.5	13.9	26.0	1.4	0.001	0.001

^1^ Contrasts: AT, TN-C vs. HS-C; PHY, HS-C vs. HS-PHY.

**Table 4:** Apparent ileal digestibilities of crude protein (CP); essential (EAAs) and non-essential amino acids (NEAAs) of thermal neutral or heat-stressed pigs offered the control diet either without or with the phytogenic solution.

	Treatment ^1^					Contrast *p*-Value ^2^		
Item	TN-C	TN-PHY	HS-C	HS-PHY	SEM	AT	PHY	AT-PHY
Crude protein	93.18	93.45	91.38	90.44	0.78	0.020	0.709	0.512
EAA								
Arginine	90.31	90.36	87.60	85.23	1.16	0.025	0.337	0.320
Histidine	88.95	89.23	86.32	84.84	1.45	0.033	0.687	0.555
Isoleucine	85.23	86.66	82.12	80.15	1.74	0.033	0.896	0.412
Leucine	85.88	87.12	82.87	81.23	1.64	0.036	0.914	0.458
Lysine	87.30	88.61	85.56	83.36	1.57	0.074	0.808	0.344
Methionine	89.54	90.43	87.42	86.23	1.33	0.063	0.926	0.512
Phenylalanine	86.58	87.69	83.29	81.84	1.54	0.023	0.923	0.481
Threonine	81.32	83.08	77.83	75.57	2.21	0.045	0.921	0.440
Tryptophan	85.56	87.29	82.15	79.88	1.68	0.015	0.888	0.314
Valine	82.99	84.38	79.22	77.00	2.01	0.032	0.859	0.447
NEAA								
Alanine	82.6	88.2	86.7	85.5	2.1	0.745	0.317	0.125
Aspartate	51.8	31.9	26.0	16.3	12.1	0.113	0.246	0.680
Cysteine	92.0	98.9	98.5	98.4	2.0	0.135	0.110	0.100
Glutamate	75.5	55.6	50.7	45.6	7.4	0.037	0.384	0.632
Glycine	82.7	88.7	85.5	82.9	2.0	0.504	0.446	0.070
Proline	83.9	80.3	73.1	71.4	3.1	0.013	0.449	0.790
Serine	82.7	80.4	74.3	71.1	2.6	0.010	0.364	0.887
Tyrosine	93.2	99.7	99.6	99.6	1.9	0.127	0.123	0.121

^1^ TN-C, thermal neutral pigs, control diet; TN-PHY, thermal neutral pigs, phytogenic solution added to diet; HS-C, heat-stressed pigs, control diet; HS-PHY, heat-stressed pigs, phytogenic solution added to diet. ^2^ Contrasts: AT, effect of AT (TN-C and TN-PHY vs. HS-C and HS-PHY); PHY, effect of supplemental PHY (TN-C and HS-C vs. TN-PHY and HS-PHY); ATxPHY, interaction of AT and PHY.

## Data Availability

The data presented in this study are available on request from the corresponding author.
